# Blood lead level is associated with non-alcoholic fatty liver disease in the Yangtze River Delta region of China in the context of rapid urbanization

**DOI:** 10.1186/s12940-017-0304-7

**Published:** 2017-08-31

**Authors:** Hualing Zhai, Chi Chen, Ningjian Wang, Yi Chen, Xiaomin Nie, Bing Han, Qin Li, Fangzhen Xia, Yingli Lu

**Affiliations:** Institute and Department of Endocrinology and Metabolism, Shanghai Ninth People’s Hospital, Shanghai JiaoTong University School of Medicine, Shanghai, 200011 China

**Keywords:** Lead, Non-alcoholic fatty liver disease, Urbanization, Chinese

## Abstract

**Background:**

China has undergone rapid urbanization in the past three decades. We aimed to report blood lead level (B-Pb) in the most rapidly urbanized Yangtze River Delta Region of China, and explore the association B-Pb and non-alcoholic fatty liver disease (NAFLD).

**Methods:**

Our data source was the SPECT-China study. We enrolled 2011 subjects from 6 villages in the Yangtze River Delta Region. Lead was measured by atomic absorption spectrometry. According to abdominal ultrasound, residents were divided into normal and NAFLD groups.

**Results:**

In total, 824 (41.0%) were diagnosed with NAFLD. Medians (interquartile range) of B-Pb were 5.29 μg/dL (3.60–7.28) [0.25 μmol/L (0.17–0.35)] for men and 4.49 μg/dL (2.97–6.59) [0.22 μmol/L (0.14–0.32)] for women. In both genders, the NAFLD group had significantly greater B-Pb than normal group (both *P* < 0.001). The prevalence of NAFLD significantly increased with increasing B-Pb quartiles in men (*P* for trend = 0.032) and women (*P* for trend = 0.001). Residents in Shanghai had significantly greater B-Pb (*P* < 0.001) and a higher prevalence of NAFLD (*P* < 0.001). Compared with women in the lowest quartile of BLL, OR of NAFLD in women in the highest quartile was 1.613 (95%CI 1.082–2.405) (*P* for trend = 0.019) after multivariable adjustment. In men, this association showed marginal significance (OR 2.168, 95%CI 0.989–4.750, *P* for trend = 0.063).

**Conclusion:**

B-Pb in Chinese residents in the Yangtze River Delta Region were much higher than in developed countries. Elevated B-Pb was associated with an increased risk of NAFLD, especially in women.

**Electronic supplementary material:**

The online version of this article (10.1186/s12940-017-0304-7) contains supplementary material, which is available to authorized users.

## Background

The prevalence of non-alcoholic fatty liver disease (NAFLD) is rising globally along with its associated conditions: obesity, dyslipidemia, insulin resistance and metabolic syndrome [1, 2]. A study from China reported that up to 42% of adults are affected by NAFLD [3]. The rise in the prevalence of NAFLD parallels with increased exposure to endocrine disruptors or endocrine-disrupting chemicals [4].

Recent evidence has gradually shown that differential exposures to environmental toxicants may play a role in the pathogenesis of NAFLD. Data from the National Health and Nutrition Examination Survey III suggested that environmental cadmium exposure was associated with NAFLD in men [5]. Another study also reported that exposure to polychlorinated biphenyls in addition to heavy metals, such as lead and mercury was associated with suspected NAFLD [6].

Lead is a nonessential xenobiotic that is considered an endocrine disrupting chemical. For decades, leaded gasoline was the dominant source of human exposure to lead. Although the Chinese government banned the use of leaded gasoline in 2000, the burgeoning lead-acid battery industry [7], the emerging e-waste recycling activities [8] and metal smelting [9] have become other large anthropogenic sources of lead pollution. Lead can be airborne or deposited on objects and can enter the human body via the ingestion of contaminated food and water, the inhalation of dust, and dermal contact [10].

Regardless of the route of exposure, the absorbed lead is conjugated in the liver, which is considered the largest lead repository and the target organ for its toxic effects [11–13]. Experimental studies have shown that chronic lead exposure can cause an elevation in alanine aminotransferase (ALT), aspartate aminotransferase, and alkaline phosphatase [14, 15]. Epidemiologic studies exploring the association between blood lead level (B-Pb) and NAFLD are scarce. Using data from NHANES 2003–2004, Cave et al. [6] reported that B-Pb was positively associated with suspected NAFLD after adjustment for age, race, sex, body mass index, poverty income ratio, and insulin resistance. However, no study has explored the association between B-Pb and NAFLD in Chinese adults so far.

Traditionally, Chinese society is divided into a rural-urban dual system based on economic and cultural disparities [16]. Compared to their rural counterparts, urban Chinese residents tend to have more educational and medical resources, better social welfare programs and higher-paying jobs [17]. Over the past three decades, the Chinese government has launched the largest-scale urbanization in human history. The Yangtze River Delta Region is located on the east coast of China, consisting of two provinces (Jiangsu, Zhejiang) and one municipality (Shanghai). During the past 30 years, this region has experienced a remarkable period of population growth and accelerated urbanization and is now the most rapidly urbanized region in China (China City Statistical Year Book, 2014). However, rapid urbanization may also cause serious environmental pollution and an increasing prevalence of non-communicable diseases, posing challenges to sustainable development in this region [18, 19]. Based on the investigation conducted by the Ministry of Land and Resources of China in 2014, approximately 19.4% of the agricultural soils have exceeded the national standards for lead, and among them the Yangtze River Delta Region is one of the most seriously polluted areas [20].

The 2014 **S**urvey on **P**revalence in **E**ast **C**hina for Me**t**abolic Diseases and Risk Factors (SPECT-China, 2014) measured the B-Pb in a Chinese population. Using these data, we aimed to explore the current B-Pb in the Yangtze River Delta Region, and further investigate the association between B-Pb and NAFLD.

## Methods

### Study population

**Fig. 1:**
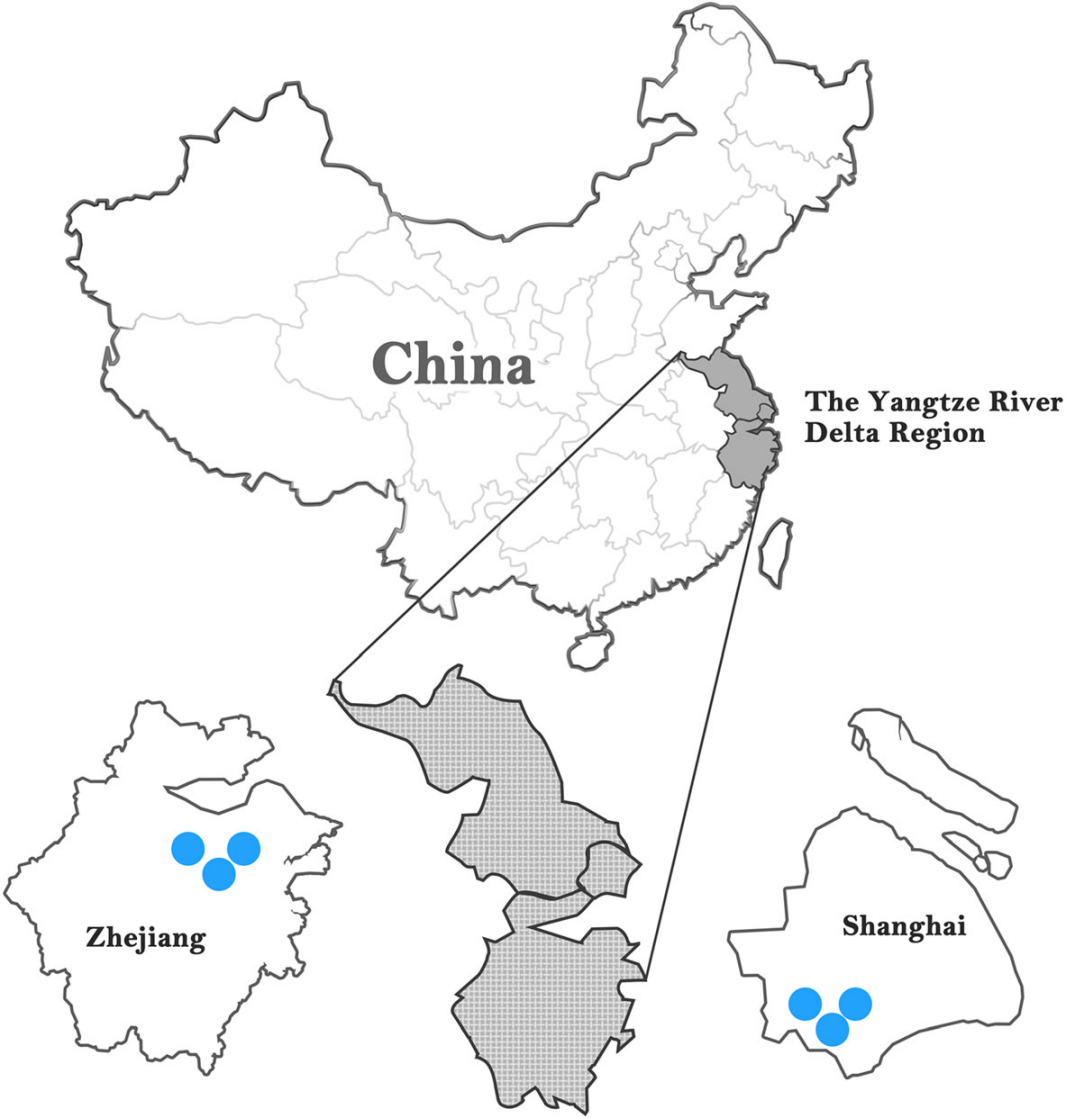
Locations of survey sites in the Yangtze River Delta region of China

The Yangtze River Delta region is the richest and most urbanized area in China. Taking in the belt of land abutting the Yangtze River, it covers 1.13% of the total land of China but supports 7.06% of the nation’s population (96.05 million) [18]. Benefiting from rapid economic development and fast-going urbanization, this region accounted for 17.19% of the nation’s Gross Domestic Product in 2013 [18]. The urbanization level of this region increased staggeringly from 20.55% in 1980 to 72.23% in 2013, resulting in a growth rate of 1.41%, which is 1.34 times the national average of 0.92% (China City Statistical Year Book, 2014). SPECT-China is a population-based cross-sectional survey on the prevalence of metabolic diseases and risk factors in East China from February to June 2014 (ChiCTR-ECS-14005052, www.chictr.org.cn) [21–23], in which six villages of the Yangtze River Delta Region were randomly chosen (Fig 1). Three villages were randomly chosen from the Fengcheng community, which is located in Southern Shanghai, 40 km away from the downtown. The other three villages were randomly chosen from the Xiaoyue community, which is located in eastern Shaoxing city (Zhejiang) adjacent to Hangzhou Bay, 45 km away from downtown. With rapid urbanization of the Yangtze River Delta region, residents living in these areas have experienced a rapid shift from peasant to citizen. Citizens ≥18 years old who had lived in their current area for at least 6 months were selected and invited to participate in the study. Those with severe communication problems, acute illness or those who were unwilling to participate were excluded from the study The overall response rate was 90.8% [21]. A total of 3427 subjects were enrolled from 3 villages in Shanghai and 3 villages in the Zhejiang province, among whom 2557 participants had no history of excessive alcohol consumption (male > 20 g/d, female >10 g/d) and viral hepatitis (self-reported). Exclusion criteria included the following: missing abdominal ultrasound (US) results (*n* = 59), missing values of ALT (*n* = 1) and B-Pb (*n* = 486). After exclusions, the study included a total number of 2011 subjects with a mean ± SD age of 54 ± 13 years.

The study protocol was approved by the Ethics Committee of the Shanghai Ninth People’s Hospital, Shanghai Jiaotong University School of Medicine. All procedures followed were in accordance with the ethical standards of the responsible committee on human experimentation (institutional and national) and with the Helsinki Declaration of 1975, as revised in 2008. All participants provided informed written consents before data collection.

#### Anthropometric and laboratory measurements

At every study site, the same trained staff collected the information on socio-demographic characteristics, medical history, and lifestyle-related risk factors using a pre-tested questionnaire. Current smoking was defined as having smoked at least 100 cigarettes in one’s lifetime and currently smoking cigarettes [24]. Standing height and body weight were measured with light clothing and no shoes. BMI was calculated as the weight in kilograms divided by the height in meters squared. Waist circumference (WC) was measured on the mid-axillary line at the midpoint between the lower border of the rib cage and the upper margin of the iliac crest.

Venous blood samples were drawn after an overnight fast of at least 8 h from all subjects. The blood samples for the fasting plasma glucose (FPG) assessment were centrifuged at the site of collection within 1 h of collection. Other blood samples were shipped in dry ice within 2–4 h of collection to a central laboratory, which was certified by the College of American Pathologists. B-Pb was determined by atomic absorption spectrometry (BH2200, China). Standard curves were established with *r* > 0.9950, and quality control materials were tested before samples were measured. Two quality control personnel participated in the process control. Outliers were detected by duplicate runs. The detection limits for blood lead were 0.1 μg/L. None of the samples exhibited values below the detection limits of blood lead. The inter-assay coefficient of variation for lead was 10%. Glycated hemoglobin (HbA1C) was assessed by high-performance liquid chromatography (MQ-2000PT, China). FPG, total cholesterol, triglycerides, high density lipoprotein, low-density lipoprotein and ALT were detected using standard laboratory methods. The laboratory methods were consistent throughout the study period. All blood samples were tested using an auto-analyzer at the central laboratory (BECKMAN COULTER AU 680 Germany).

#### Definition of variables

An abdominal ultrasonic (US) examination was performed on all participants using a B-mode ultrasound device (MINDRAY M7, China) by two trained US doctors who were blinded to the clinical and laboratory data. According to the criteria described previously [25, 26], the ultrasonographic findings were categorized into two patterns: normal, homogenous liver parenchyma with medium-level echogenicity and a regular hepatic surface; and fatty liver, discrete coarse and heterogeneous parenchymal echogenicity and dotted, irregular or nodular hepatic liver surface. In accordance with the American Diabetes Association 2014 criteria, diabetes was defined as a previous diagnosis by healthcare professionals, FPG ≥7.0 mmol/L, or HbA1c ≥6.5%.

#### Statistical analysis

All data analyses were performed with IBM SPSS Statistics, Version 22 (IBM Corporation, Armonk, NY, USA). Two-sided *P* values < 0.05 were considered significant. General characteristics were summarized as the median and interquartile range for continuous variables or as the number and proportion for categorical variables. To test for differences in variables among different groups, the Kruskal-Wallis test was used for continuous variables with a skewed distribution, and the Pearson chi-square test was used for categorical variables.

To determine the risk of NAFLD for each quartile of B-Pb, the binary logistic regression analysis was applied with the lowest B-Pb quartile as reference. The data were expressed as odds ratio (OR) and 95% confidence intervals (CIs). Model 1 was unadjusted. Model 2 included terms for age, geography (Shanghai/Zhejiang), educational level, current smoking, current drinking and ALT. Model 3 was further adjusted for diabetes, WC, BMI, low-density lipoprotein, high-density lipoprotein, triglycerides, total cholesterol and cadmium. WC was adjusted, because it may be better than body mass index as an alternative measure of body fat deposition for predicting cardio-metabolic risks [27, 28]. Moreover, among Chinese adults, people are more likely to have generally low BMI but visceral adiposity [29].

## Results

General demographic and laboratory characteristics of the study population categorized by the presence/absence of NAFLD are presented in Table 1. Overall, the prevalence of NAFLD was 41.0% in this study population (40.8% in men and 41.0% in women). Medians (IQR) of B-Pb were 5.29 μg/dL (3.60–7.28) [0.25 μmol/L (0.17–0.35)] for men and 4.49 μg/dL (2.97–6.59) [0.22 μmol/L (0.14–0.32)] for women. Compared to subjects without NAFLD, both men and women with NAFLD had significantly higher B-Pb levels (men: 5.65 (4.00–7.76) vs 5.12 (3.30–6.90) μg/dL [0.27 (0.19–0.37) vs 0.25 (0.16–0.33) μmol/L], *P* = 0.006; women: 4.80 (3.20–6.94) vs 4.24 (2.80–6.24) μg/dL [0.23 (0.15–0.33) vs 0.20 (0.13–0.30) μmol/L], *P* < 0.001). Moreover, subjects of both genders with NAFLD had a greater ALT and WC, a worse lipid profile, and a higher prevalence of diabetes.

**Table 1:** Demographic and general characteristics of the study participants

	Men			Women		
	Non-NAFLD	NAFLD	*P*	Non-NAFLD	NFALD	*P*
N	311	214		876	610	
Age, yr	56 (44–64)	57 (44–66)	0.723	51 (41–61)	59 (51–64)	<0.001
Blood lead level, μg/dL	5.12 (3.30–6.90)	5.65 (4.00–7.76)	0.006	4.24 (2.80–6.24)	4.80 (3.20–6.94)	<0.001
Blood cadmium level, μg/dL	0.15 (0.05–0.32)	0.21 (0.08–0.36)	0.017	0.14 (0.05–0.31)	0.16 (0.06–0.31)	0.295
ALT, U/L	19.0 (15.0–26.0)	24.0 (18.0–34.0)	<0.001	15.0 (12.0–20.0)	18.0 (15.0–25.0)	<0.001
Educational level, %			0.380			0.001
<High School	76.0	77.9		83.9	90.5	
High school	15.4	16.8		10.7	7.5	
>High School	8.6	5.3		5.4	2.0	
Waist circumference, cm	76.5 (72.0–83.0)	86.0 (82.0–92.0)	<0.001	72.0 (68.0–78.0)	83.0 (77.0–89.0)	<0.001
Body mass index, kg/m^2^	22.5 (20.8–24.5)	26.1 (23.8–28.0)	<0.001	22.4 (20.4–24.4)	26.0 (24.2–28.4)	<0.001
LDL-cholesterol, mmol/L	2.67 (2.26–3.09)	2.92 (2.46–3.38)	<0.001	2.63 (2.24–3.09)	2.97 (2.54–3.43)	<0.001
HDL-cholesterol, mmol/L	1.39 (1.18–1.58)	1.22 (1.08–1.40)	<0.001	1.51 (1.32–1.71)	1.39 (1.23–1.58)	<0.001
Triglycerides, mmol/L	1.10 (0.86–1.49)	1.66 (1.19–2.35)	<0.001	1.06 (0.81–1.46)	1.48 (1.08–2.14)	<0.001
Total-cholesterol, mmol/L	4.84 (4.25–5.41)	5.09 (4.56–5.68)	0.001	4.87 (4.33–5.48)	5.27 (4.58–5.93)	<0.001
Diabetes, %	9.7	15.0	0.002	5.4	19.7	0.001
Current smoker, %	47.1	49.0	0.681	1.8	2.3	0.524

Data were summarized as median with interquartile range for continuous variables or as number with proportion for categorical variables

*NAFLD* non-alcoholic fatty liver disease, *ALT* alanine aminotransferase, *LDL* low-density lipoprotein, *HDL* high-density lipoprotein

The characteristics of the study population according to quartiles of B-Pb are summarized in Table 2. Compared with those in the lowest quartile, subjects of both genders in the highest quartile were older and more likely to have lower educational levels (*P* < 0.05). Additionally, with increasing B-Pb quartiles, women had a significantly greater WC and BMI and a worse lipid profile (*P* for trend <0.05). However, such trend was not observed across the B-Pb quartiles in men (*P* for trend > 0.05).

Additional file 1: Tables S1 and S2 summarize the results of B-Pb and metabolic factors by geography. Compared with those living in Zhejiang, residents in Shanghai had significantly higher B-Pb (5.54 (4.03–7.78) vs 3.55 (2.50–5.30) μg/dL [0.27 (0.19–0.37) vs 0.17 (0.12–0.25) μmol/L], *P* < 0.001), and also a higher prevalence of NAFLD (45.7% vs 35.1%, *P* < 0.001).

**Table 2:** General characteristics of the study population by blood lead quartiles

	Quartile 1	Quartile 2	Quartile 3	Quartile 4	*P* for trend
Men					
N	133	130	131	131	
Blood lead level, μg/dL	≤3.60	3.61–5.29	5.30–7.28	≥7.29	
Age, yr	52 (39–61)	53 (42–64)	59 (48–66)	60 (51–69)	<0.001
Blood cadmium level, μg/dL	0.07 (0.04–0.23)	0.16 (0.07–0.29))	0.19 (0.08–0.33)	0.30 (0.12–0.45)	<0.001
Educational level, %					<0.001
< High School	69.3	69.6	84.5	82.5	
High school	17.5	21.4	12.2	13.3	
> High School	13.2	9.0	3.3	4.2	
ALT, U/L	21.0 (17.0–33.0)	20.0 (16.0–27.0)	21.0 (16.0–28.0)	20.0 (15.0–28.0)	0.062
Waist circumference, cm	80.0 (74.0–86.0)	80.0 (73.0–86.0)	80.0 (75.0–87.0)	84.0 (76.0–90.0)	0.029
Body mass index, kg/m^2^	24.5 (21.6–26.3)	23.6 (21.1–26.0)	23.7 (21.7–26.0)	24.1 (22.0–27.3)	0.157
LDL-cholesterol, mmol/L	2.81 (2.30–3.15)	2.66 (2.31–3.10)	2.74 (2.33–3.25)	2.90 (2.34–3.29)	0.206
HDL-cholesterol, mmol/L	1.34 (1.17–1.54)	1.34 (1.09–1.53)	1.28 (1.10–1.49)	1.32 (1.14–1.52)	0.340
Triglycerides, mmol/L	1.30 (0.90–1.85)	1.28 (0.96–1.74)	1.33 (0.93–1.99)	1.28 (0.97–1.90)	0.817
Total-cholesterol, mmol/L	4.92 (4.37–5.56)	4.84 (4.31–5.43)	4.93 (4.26–5.47)	4.95 (4.38–5.60)	0.694
Diabetes, %	12.0	7.7	12.3	15.3	0.017
Current smoker, %	46.5	54.5	52.8	39.0	0.076
Women					
N	372	371	372	371	
Blood lead level, μg/dL	≤2.97	2.98–4.49	4.50–6.59	≥6.60	
Age, yr	52 (41–60)	53 (43–62)	54 (45–63)	59 (50–66)	<0.001
Blood cadmium level, μg/dL	0.06 (0.03–0.13)	0.13 (0.06–0.27)	0.18 (0.07–0.34)	0.27 (0.14–0.44)	<0.001
Educational level, %					0.001
< High School	84.1	82.2	89.1	90.9	
High school	10.8	12.2	7.3	7.3	
> High School	5.1	5.6	3.6	1.8	
ALT, U/L	17.0 (13.0–22.0)	17.0 (13.0–23.0)	16.0 (13.0–22.0)	16.0 (13.0–20.0)	0.930
Waist circumference, cm	75.0 (70.0–82.0)	76.0 (70.0–82.0)	76.0 (70.0–84.3)	79.0 (72.0–85.0)	<0.001
Body mass index, kg/m^2^	23.2 (21.0–25.6)	23.7 (21.2–25.8)	24.0 (21.7–26.4)	24.8 (22.5–27.9)	<0.001
LDL-cholesterol, mmol/L	2.61 (2.24–3.18)	2.68 (2.28–3.16)	2.80 (2.35–3.31)	2.94 (2.56–3.40)	<0.001
HDL-cholesterol, mmol/L	1.47 (1.28–1.63)	1.47 (1.27–1.66)	1.45 (1.29–1.67)	1.47 (1.29–1.68)	0.118
Triglycerides, mmol/L	1.18 (0.83–1.58)	1.21 (0.88–1.69)	1.21 (0.92–1.71)	1.27 (0.95–1.82)	0.03
Total-cholesterol, mmol/L	4.91 (4.33–5.66)	4.88 (4.36–5.55)	5.04 (4.47–5.64)	5.18 (4.55–5.78)	0.004
Diabetes, %	8.3	11.1	11.0	14.6	0.20
Current smoker, %	3.6	0.8	1.7	1.7	0.147

Data were summarized as median with interquartile range for continuous variables or as number with proportion for categorical variables

*ALT* alanine aminotransferase, *LDL* low-density lipoprotein, *HDL* high-density lipoprotein

**Fig. 2:**
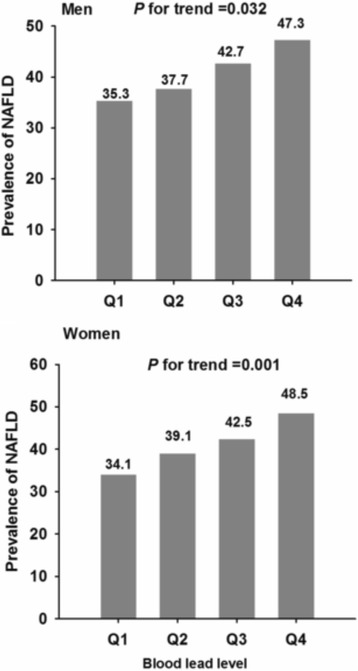
The prevalence of non-alcoholic fatty liver disease according to blood lead level quartiles

The prevalence of NALFD in participants according to B-Pb quartiles are presented in Fig. 2. The prevalence of NAFLD gradually and markedly increased with increasing B-Pb quartiles in both men (*P* for trend = 0.032) and women (*P* for trend =0.001).

Table 3 demonstrates the results of binary logistic regression analyses measuring the association of B-Pb with the risk for NAFLD. In the unadjusted model, compared to those in the lowest B-Pb quartile, the ORs of NAFLD in the highest quartile were 1.644 (95% CI 1.003, 2.695) for men and 1.818 (95% CI 1.353, 2.443) for women (Table 3, Model 1). Adjusting for socioeconomic variables and ALT did not weaken the association between B-Pb and NAFLD in either gender (Table 3, Model 2). Further adjustment for diabetes, WC, BMI, lipid profile and cadmium weakened this association such that it was only marginally significant in men [OR 2.168 (95% CI 0.989–4.750), *P* for trend = 0.063); however, in women this association remained statistically significant [OR 1.613 (95% CI 1.082, 2.405), *P* for trend =0.019].

**Table 3:** Association of blood lead level with non-alcohol fatty liver disease

	Blood lead level quartiles				
Variables	Q1	Q2	Q3	Q4	P for trend
*Men*					
Blood lead level, μg/dL	≤3.60	3.61–5.29	5.30–7.28	≥7.29	
NAFLD					
Model 1	Ref.	1.107 (0.670–1.829)	1.366 (0.832–2.244)	1.644 (1.003–2.695)*	0.033
Model 2	Ref.	1.261 (0.695–2.287)	1.394 (0.770–2.523)	1.858 (1.007–3.429)*	0.048
Model 3	Ref.	1.695 (0.841–3.419)	1.837 (0.875–3.858)	2.168 (0.989–4.750)	0.063
*Women*					
Blood lead level, μg/dL	≤2.97	2.98–4.49	4.50–6.59	≥6.60	
NAFLD					
Model 1	Ref.	1.238 (0.918–1.669)	1.424 (1.058–1.917)*	1.818 (1.353–2.443)***	<0.001
Model 2	Ref.	1.276 (0.915–1.779)	1.585 (1.140–2.204)**	1.686 (1.211–2.348)**	0.001
Model 3	Ref.	1.384 (0.956–2.005)	1.495 (1.024–2.181) *	1.613 (1.082–2.405)*	0.019

Data were odds ratio (95% confidence interval). ^*^ *P* < 0.05; ^**^ *P* < 0.01; ^***^ *P* < 0.001

Model 1 was unadjusted

Model 2 was adjusted for age, geography (Shanghai/Zhejiang), educational level, current smoking, current drinking and alanine transaminase

Model 3 was additionally adjusted for diabetes, waist circumference, body mass index, low-density lipoprotein, high-density lipoprotein, triglycerides, total cholesterol and blood cadmium level

## Discussion

To the best of our knowledge, this study reported B-Pb in the largest sample of the general Chinese population living in the rapidly urbanized Yangtze River Delta Region. This study is also the first to explore the association between B-Pb and NAFLD in Chinese adults. We found that B-Pb was positively associated with NAFLD. This association was independent of liver function (ALT) and conventional NAFLD risk factors including lifestyle, WC, BMI, lipid profile, diabetes and cadmium.

China has been experiencing a period of accelerated urbanization since the 1990s. The Yangtze River Delta, the study area of the present paper, boasts a faster growth rate of urbanization than the national average level (China City Statistical Year book, 2014). Rapid urbanization results in changes in the lifestyles of residents [30]. Growing numbers of people have become reliant on automobiles and can afford computers, displacing vigorous physical activity with a sedentary lifestyle. Meanwhile, the consumption of low-value high-calorie options such as fast food has increased. A sedentary lifestyle and increased intake of calories are known risk factors for obesity, NAFLD and other metabolic diseases [31].

Urbanization not only encourages lifestyle changes but also causes changes in the urban environment, including air pollution and noise induced by construction and transportation, and soil and water pollution from waste disposal [32]. Based on the investigation conducted by the Ministry of Land and Resources of China in 2014, approximately 19.4% of the agricultural soils have exceeded the national standards for lead, and among them the Yangtze River Delta Region is one of the most seriously polluted areas [20]. Emerging evidence has shown that in addition to conventional risk factors, various environmental factors termed endocrine-disrupting chemicals can have an additive or synergistic effect on metabolic disorders [33].

Scarce epidemiologic studies have explored the association between lead exposure and NAFLD. In 2010, Cave et al. [6] reported that B-Pb (median = 1.60 μg/dL) were positively associated with suspected NAFLD in 4582 participants based on data from the NHANES 2003–2004 [OR 1.6 (95% CI 1.1, 2.3)]. To date, this study is the only epidemiologic investigation detecting an association between the B-Pb and NAFLD. However, it is notable that in the NHANES study, NAFLD was defined by an elevation in ALT that was not attributable to viral hepatitis, hemochromatosis, or alcoholism. Considering that the ALT level may be normal in NAFLD and that there are many causes of increased ALT other than NAFLD, this definition has limitations. In contrast, we used ultrasound to screen for fatty liver and categorized the degree of fat accumulation into normal and fatty liver groups, which has a sensitivity of 94% and a specificity of 84% for detecting liver steatosis [34].. Additionally, compared with Cave et al.’s analysis, this study controlled for age, sex, BMI and current smoking, but also made additional adjustments for ALT, waist circumference, diabetes and lipid profile. Thus, the results from this analysis of B-Pb and NAFLD may be more precise. Finally, the study by Cave et al. did not perform sex-specific analyses, though sex was adjusted.

The mechanism underlying the association between B-Pb and NAFLD is not yet fully understood. First, oxidative stress may be involved. Lead exposure causes alterations in lipid peroxidation, overproduction of reactive oxygen species, and reduction in the activity of antioxidant enzymes, such as superoxide dismutase, catalase, and glutathione peroxidase in hepatocytes [14, 35–37]. Recent studies have shown that oxidative stress is related to the pathogenic mechanism of NAFLD [38]. Thus, it is reasonable to deduce that lead exposure may induce NAFLD partly through oxidative stress. In addition, lead stimulates intercellular signaling between hepatocytes and Kupffer cells, which contributes to lead-induced hepatotoxicity [39].

Accumulating evidence has shown that there is no safe threshold for B-Pb [40]. Although the B-Pb has gradually dropped in the past decades [41], the median B-Pb (4.71 μg/dL [0.23 μmol/L]) in Chinese residents in the rapidly urbanized Yangtze River Delta Region was still much higher than that in Americans (0.84 μg/dL [0.04 μmol/L]) based on the newest data from NHANES 2013–2014 [42]. Considering the fact that China’s urbanization rate continues to move forward, our study may have important implications from a public health perspective. It is now time to pay attention to urbanization quality rather than to continue large-scale “destroy and build.” Large numbers of people are experiencing remarkable lifestyle and environmental changes during the transition of urbanization. They should be encouraged to adopt healthy lifestyles, and more importantly, to participate in environmental monitoring and management.

Our study has some strengths. First, we have provided the newest data regarding the B-Pb in a large population that has experienced rapid urbanization over the past three decades. This study is also the first to explore the association between B-Pb and NAFLD in Chinese adults. Second, our study was a community-dwelling population-based design, with a wide spectrum of information on confounders, so our results are more representative in comparison to a clinic-based population. However, our study also has some limitations. First, in light of the cross-sectional nature of this study, we cannot infer any cause-effect relationships between B-Pb and NAFLD. The B-Pb concentration may be affected by liver function. However, we have already adjusted ALT in the logistic regression models. Even we reanalyzed the model within normal ALT level, the association between B-Pb and NAFLD remained robust. Prospective studies are needed to confirm our findings. Second, the use of liver ultrasound to diagnose NAFLD is operator-dependent. However, our US examinations were performed by the same two operators by consensus to minimize the deviation. Liver biopsy, the current gold standard for diagnosing NAFLD, was not possible in such a large epidemiological study.

## Conclusions

We have demonstrated that an elevated B-Pb was associated with an increased prevalence of NAFLD in a Chinese population undergoing rapid urbanization. Future prospective studies are warranted to confirm our findings.

## Additional file

Additional file 1: Supplementary Tables. **Table S1** Demographic and general characteristics of the study participants. **Table S2** General characteristics of the study population by blood lead quartiles. (DOC 101 kb)

## Abbreviations

ALT: Alanine aminotransferase
BMI: Body mass index
B-Pb: Blood lead level
CI: Confidence interval
LDL-C: Low-density lipoprotein cholesterol
NAFLD: Non-alcoholic fatty liver disease
NHANES: National Health and Nutrition Examination Survey
OR: Odds ratio
WC: Waist circumference
