# Supplementary material for: Blood lead level is associated with non-alcoholic fatty liver disease in the Yangtze River Delta region of China in the context of rapid urbanization
Source: Environ Health. 2017 Aug 31;16:93. doi: 10.1186/s12940-017-0304-7 (PMC5580229; doi:10.1186/s12940-017-0304-7)
Supplement: Additional file 1: — Supplementary Tables. Table S1 Demographic and general characteristics of the study participants. Table S2 General characteristics of the study population by blood lead quartiles. (DOC 101 kb) [file 12940_2017_304_MOESM1_ESM.doc]

Table 1 Demographic and general characteristics of the study participants

|  | Zhejiang | |  | Shanghai | |  |
| --- | --- | --- | --- | --- | --- | --- |
|  | Non-NAFLD | NAFLD | *P* | Non-NAFLD | NFALD | *P* |
| N | 578 | 312 |  | 609 | 512 |  |
| Age, yr | 50 (40-61) | 59 (48-65) | <0.001 | 53 (44-62) | 59 (50-65) | <0.001 |
| Men, n (%) | 158 (27.3) | 92 (29.5) | 0.496 | 153 (25.1) | 122 (23.8) | 0.616 |
| Blood lead level, μg/dL | 3.39 (2.40-5.11) | 3.80 (2.63-5.62) | 0.025 | 5.40 (3.91-7.54) | 5.83 (4.23-8.08) | 0.016 |
| ALT, U/L | 17.0 (13.0-23.0) | 21.0 (16.0-32.0) | <0.001 | 15.0 (12.0-20.0) | 19.0 (15.0-25.0) | <0.001 |
| Educational level, % |  |  | <0.001 |  |  | 0.187 |
| <High School | 82.4 | 95.0 |  | 81.4 | 83.2 |  |
| High school | 10.8 | 3.1 |  | 12.9 | 13.5 |  |
| >High School | 6.8 | 1.9 |  | 5.7 | 3.3 |  |
| Waist circumference, cm | 72.0 (67.0-77.0) | 83.0 (78.0-88.0) | <0.001 | 75.0 (70.0-81.0) | 84.0 (78.0-91.0) | <0.001 |
| Body mass index, kg/m² | 21.4 (19.8-23.3) | 25.3 (23.7-27.2) | <0.001 | 23.5 (21.5-25.3) | 26.8 (24.5-28.8) | <0.001 |
| LDL-cholesterol, mmol/L | 2.47 (2.14-2.89) | 2.80 (2.32-3.22) | <0.001 | 2.81 (2.38-3.30) | 3.05 (2.64-3.54) | <0.001 |
| HDL-cholesterol, mmol/L | 1.49 (1.29-1.67) | 1.34 (1.19-1.52) | <0.001 | 1.49 (1.28-1.71) | 1.35 (1.18-1.54) | <0.001 |
| Triglycerides, mmol/L | 1.04 (0.78-1.40) | 1.54 (1.08-2.12) | <0.001 | 1.10 (0.87-1.55) | 1.53 (1.13-2.21) | <0.001 |
| Total-cholesterol, mmol/L | 4.79 (4.27-5.39) | 5.15 (4.42-5.79) | <0.001 | 4.91 (4.34-5.53) | 5.24 (4.65-5.90) | <0.001 |
| Diabetes, % | 6.2 | 17.6 | <0.001 | 6.7 | 18.9 | <0.001 |
| Current smoker, % | 15.7 | 19.5 | 0.158 | 14.3 | 11.7 | 0.211 |

Data were summarized as median with interquartile range for continuous variables or as number with proportion for categorical variables.

NAFLD, non-alcoholic fatty liver disease; ALT, alanine aminotransferase; LDL, low-density lipoprotein; HDL, high-density lipoprotein.

Table 2 General characteristics of the study population by blood lead quartiles

|  | Quartile 1 | Quartile 2 | Quartile 3 | Quartile 4 | *P* for trend |
| --- | --- | --- | --- | --- | --- |
| **Zhejiang** |  |  |  |  |  |
| N | 229 | 216 | 226 | 219 |  |
| Blood lead level, μg/dL | ≤2.50 | 2.51-3.55 | 3.56-5.30 | ≥5.31 |  |
| Age, yr | 52 (40-60) | 52 (43-62) | 53 (43-64) | 58 (46-66) | <0.001 |
| Educational level, % |  |  |  |  | 0.172 |
| <High School | 84.8 | 84.8 | 88.6 | 88.6 |  |
| High school | 9.1 | 9.2 | 7.3 | 7.1 |  |
| >High School | 6.1 | 6.0 | 4.1 | 4.3 |  |
| ALT, U/L | 18.0 (14.0-25.5) | 19.0 (14.0-27.0) | 18.0 (14.0-28.0) | 18.0 (14.0-26.0) | 0.861 |
| Waist circumference, cm | 76.0 (70.0-83.0) | 76.0 (70.0-82.0) | 76.0 (70.0-83.0) | 76.0 (70.0-82.0) | 0.702 |
| Body mass index, kg/m² | 23.1 (20.6-25.4) | 22.6 (20.6-25.1) | 22.7 (20.4-25.2) | 22.8 (20.5-24.8) | 0.206 |
| LDL-cholesterol, mmol/L | 2.50 (2.24-3.01) | 2.50 (2.13-2.92) | 2.56 (2.19-3.01) | 2.66 (2.26-3.07) | 0.09 |
| HDL-cholesterol, mmol/L | 1.45 (1.25-1.59) | 1.45 (1.23-1.64) | 1.41 (1.25-1.57) | 1.42 (1.27-1.64) | 0.501 |
| Triglycerides, mmol/L | 1.12 (0.84-1.59) | 1.18 (0.81-1.52) | 1.20 (0.84-1.64) | 1.20 (0.90-1.79) | 0.184 |
| Total-cholesterol, mmol/L | 4.82 (4.29-5.54) | 4.83 (4.23-5.47) | 4.88 (4.29-5.54) | 5.05 (4.47-5.60) | 0.045 |
| Diabetes, % | 10.5 | 10.2 | 10.2 | 10.1 | 0.602 |
| Current smoker, % | 11.8 | 14.8 | 18.3 | 23.5 | 0.001 |
| **Shanghai** |  |  |  |  |  |
| N | 281 | 280 | 280 | 280 |  |
| Blood lead level, μg/L | ≤4.03 | 4.04-5.54 | 5.55-7.78 | ≥7.79 |  |
| Age, yr | 52 (42-61) | 55 (44-62) | 56 (48-64) | 60 (51-69) | <0.001 |
| Educational level, % |  |  |  |  | <0.001 |
| <High School | 74.2 | 79.2 | 87.4 | 88.3 |  |
| High school | 17.2 | 16.0 | 9.6 | 9.8 |  |
| >High School | 8.6 | 4.8 | 3.0 | 1.9 |  |
| ALT, U/L | 16.0 (12.0-21.0) | 17.0 (13.0-22.0) | 17.0 (14.0-23.0) | 17.0 (13.0-23.0) | 0.247 |
| Waist circumference, cm | 77.0 (70.0-84.0) | 78.0 (73.0-87.0) | 80.0 (73.0-86.0) | 82.0 (74.0-89.0) | <0.001 |
| Body mass index, kg/m² | 24.4 (22.2-26.7) | 24.6 (22.4-26.9) | 25.0 (22.6-27.7) | 25.3 (23.1-27.9) | 0.002 |
| LDL-cholesterol, mmol/L | 2.97 (2.48-3.50) | 2.90 (2.44-3.38) | 2.86 (2.43-3.34) | 3.03 (2.61-3.43) | 0.948 |
| HDL-cholesterol, mmol/L | 1.43 (1.25-1.64) | 1.42 (1.22-1.64) | 1.41 (1.22-1.65) | 1.41 (1.22-1.64) | 0.83 |
| Triglycerides, mmol/L | 1.34 (0.95-1.85) | 1.28 (0.96-1.89) | 1.22 (0.94-1.81) | 1.32 (0.96-1.82) | 0.634 |
| Total-cholesterol, mmol/L | 5.11 (4.39-5.87) | 5.02 (4.47-5.65) | 5.02 (4.45-5.62) | 5.18 (4.54-5.72) | 0.433 |
| Diabetes, % | 9.3 | 9.6 | 15.0 | 15.4 | <0.001 |
| Current smoker, % | 8.6 | 11.1 | 14.8 | 18.0 | 0.001 |

Data were summarized as median with interquartile range for continuous variables or as number with proportion for categorical variables.

ALT, alanine aminotransferase; LDL, low-density lipoprotein; HDL, high-density lipoprotein.
